# Supplementary material for: Prevalence and associations of anisometropia with spherical ametropia, cylindrical power, age, and sex, based on 134,603 refractive surgery candidates
Source: PLoS One. 2025 Jan 3;20(1):e0315080. doi: 10.1371/journal.pone.0315080 (PMC11698341; doi:10.1371/journal.pone.0315080)
Supplement: Attachment — Submitted filename: Response to Reviewers.docx [file pone.0315080.s002.docx]

# *Response to Reviewers*

Dear Mr Der-Chong Tsai,

Thank you for inviting us to submit a revised draft of our manuscript entitled, "Prevalence and associations of anisometropia with spherical ametropia, cylindrical power, age, and sex, based on 134,603 refractive surgery candidates" (PONE-D-24-18058) to PLOS ONE. We also appreciate the time and effort you and each of the reviewers have dedicated to providing insightful feedback on ways to strengthen our paper. Thus, it is with great pleasure that we resubmit our article for further consideration. We have incorporated changes that reflect the detailed suggestions you have graciously provided. We also hope that our edits and the responses we provide below satisfactorily address all the issues and concerns you and the reviewers have noted.

To facilitate your review of our revisions, the following is a point-by-point response to the questions and comments delivered in your letter dated June 28, 2024:

EDITORIAL COMMENTS

*1. Please ensure that your manuscript meets PLOS ONE's style requirements, including those for file naming.*

- Response: We have ensured that our manuscript meets PLOS ONE's style requirements, including those for file naming.

*2. Please provide additional details regarding participant consent. In the ethics statement in the Methods and online submission information, please ensure that you have specified (1) whether consent was informed and (2) what type you obtained (for instance, written or verbal, and if verbal, how it was documented and witnessed). If your study included minors, state whether you obtained consent from parents or guardians. If the need for consent was waived by the ethics committee, please include this information.*

- Response: We have obtained written consent from our patients, which were fully anonymized. This is now stated in detail in lines 85 to 91.

*3. We note that your Data Availability Statement is currently as follows: [All relevant data are within the manuscript and its Supporting Information files.] Please confirm at this time whether or not your submission contains all raw data required to replicate the results of your study. Authors must share the “minimal data set” for their submission. (…)*

- Response: We now included the minimal data set necessary to replicate our study findings as a xlsx-File in the Supporting Information section.

*4. Please include your full ethics statement in the ‘Methods’ section of your manuscript file. In your statement, please include the full name of the IRB or ethics committee who approved or waived your study, as well as whether or not you obtained informed written or verbal consent. If consent was waived for your study, please include this information in your statement as well.*

- We have included the full ethics statement in the ‘Methods’ section of your manuscript file. We have obtained written consent. Cf. lines 85-91

REVIEWER 1:

*1. This study involved a study population who visited refractive clinics for refractive surgery. They were either myopic (80.8%) or hyperopic, which is different from the general population. Moreover, gender, income, and many other factors may also affect the willingness to undergo refractive surgeries. The authors should be cautious when interpreting the results and should inform the readers about the impact of selection bias. For example, the prevalence and severity of anisometropia may be overestimated within the SE range of -1.0D to +1.0D. Few isometropic subjects with SE between -1.0D and +1.0D would seek refractive surgery.*

- Response: Thank you for your valuable comment on the selection bias. We kindly refer to lines 350 to 388 with an in-depth discussion on selection bias.

*2. For the definition of anisometropia, does it include a difference of 1 Diopter (≥1D) or not (>1D)? In Line 147 and Line 163, it seems that 1D is not included as anisometropia. However, in Line 87 and Line 149, 1D is classified as mild anisometropia. In Table 1, does the symbol “[1,max]” indicate that an SE difference from 1 to the maximum value is considered anisometropia?*

- Response: A difference of 1 Diopter *(≥1D) is consistently included as anisometropia. We have corrected the typographical errors in line 87 (now line 111) and in line 149 (now line 173). Table 1 (lines 181-182) was insofar clarified (see also Point 3 below).*

*3. In Table 1, Figure 1, Figure 2, and Figure 3, please do not use parentheses “()” or brackets “[]” to indicate ranges. Instead, use symbols such as “>”, “≥”or “<” to clearly denote the ranges.*

- Response: We changed Table 1 and the Figures accordingly. In the course of adjusting the figure legends, we switched to Mild/Moderate/Severe anisometropia in line with the definition we give in the manuscript text.

*4. What are the exclusion criteria of the study? Were subjects with previous refractive surgery or cataract surgery in any eye excluded from the analysis?*

- Response: We specified the exclusion criteria (patients with a previous surgical intervention, such as cataract surgery, or patients with an eye condition) in lines 83-84.

*5. The results for hyperopic subgroups regarding the association of anisometropia with spherical ametropia and cylindrical power differ from the results of Qin et al. (2005) and Linke et al. (2011). Please compare these results.*

- Response: You have of course made an interesting and important point here. We gladly added this to the discussion and compared both aforementioned studies with our results (cf. lines 390 onwards, lines 414 onwards and lines 456 onwards in the Discussion chapter).

REVIEWER 2:

*1. Please clarify certificate approval number*

- Response: We added the number (#2021-1278) in lines 88-89.

*2. Introduction is not sufficient regarding the domain of study and what will added*

- Response: We amended our introduction and expanded on the domain of study in lines 70 to 75 in the Introduction.

*3. Please clarify inclusion and exclusion criteria in details*

- Response: We specified the inclusion criteria in lines 78-83 and furthermore exclusion criteria (patients with a previous surgical intervention, such as cataract surgery, or patients with an eye condition) in lines 83-84.

*4. Please clarify setting (study location) and study design*

- Response: We amended our Methods section (lines 77 onward) to include more details on study setting and design.

*5. Please clarify methods of examinations of visual acuity, IOP, refraction , pupil*

- Response: We amended our Methods section in lines 92-100 to include more details.

*6. Discussion is not sufficient regarding comparison with recent citations*

- Response: We included three more recent citations (2020, 2023, 2024) in lines 408-412 of our Discussion.

*7. Conclusion should be condensed*

- Response: We revised and condensed the Conclusion (cf. Lines 495-502)

*8. Please added recent references*

- Response: We added 5 recent references in total (cf. lines 51-54).

9. Please clarify consent for publications, funding, avialbility of materials and data, abbreviations, conflict of interests, standards of reporting, ORCID of authors

- Response: We clarified consent for publication (cf. lines 90-91) and included additional information on funding and competing interests in a separate ‘Additional Information’ section (cf. lines 558-565). We included the minimal data set required to replicate our study in an Excel file in the Supporting Information section. Abbreviations were consistently introduced at first mention. My ORCID number: 0000-0001-8977-4261

*10. Some typographical errors should be corrected*

- Response: We revised the manuscript and corrected remaining typographical errors.

Once again, we extend our gratitude for the valuable insights and your dedication in reviewing our manuscript. We hope that the revised version of our manuscript will meet your approval. Should you have any further questions or require additional information, please feel free to contact us.

Thank you for your time and consideration.

Sincerely,

Andreas Frings, MD

Corresponding Author

University Hospital Düsseldorf
