# Supplementary material for: Prevalence and associations of anisometropia with spherical ametropia, cylindrical power, age, and sex, based on 134,603 refractive surgery candidates
Source: PLoS One. 2025 Jan 3;20(1):e0315080. doi: 10.1371/journal.pone.0315080 (PMC11698341; doi:10.1371/journal.pone.0315080)
Supplement: Attachment — Submitted filename: Response to Reviewers.docx [file pone.0315080.s003.docx]

# *Response to Reviewers*

Dear Mr Der-Chong Tsai,

Thank you for revising our manuscript: "Prevalence and associations of anisometropia with spherical ametropia, cylindrical power, age, and sex, based on 134,603 refractive surgery candidates" (PONE-D-24-18058 / PONE-D-24-18058R1) to PLOS ONE, a second time.

We are grateful for the time and effort you and the reviewers have dedicated once again on our paper. We have made revisions based on the detailed suggestions you kindly provided and hope that our edits, along with the responses below, satisfactorily address all the concerns and issues raised.

To facilitate your review of our revisions, the following is a point-by-point response to the questions and comments delivered in your letter dated September 22, 2024:

JOURNAL REQUIREMENTS

*Please review your reference list to ensure that it is complete and correct. If you have cited papers that have been retracted, please include the rationale for doing so in the manuscript text, or remove these references and replace them with relevant current references. Any changes to the reference list should be mentioned in the rebuttal letter that accompanies your revised manuscript. If you need to cite a retracted article, indicate the article’s retracted status in the References list and also include a citation and full reference for the retraction notice.*

- Response: We corrected some publication data in line 550 and 582 next to several typographical corrections. In lines 583-584 we have added an additional reference (Discussion chapter, see reviewer comments below) so that we now consider the reference list complete and correct. We furthermore checked that no cited article has been retracted.

REVIEWER COMMENTS

*1. I recommend having the manuscript reviewed by a native English speaker to enhance the language quality.*

- Response: We had the manuscript proofread and corrected by a native English speaker.

*2. Thank you for your response. The authors have addressed most of the points. However, in the "Discussion" section, I hope the authors can provide explanations for the data rather than simply comparing the results with previous studies. In particular, I suggest elaborating on the sections regarding "selection bias" and the "associations between anisometropia and spherical ametropia." What are the potential reasons for the observed differences in data compared to previous research? Are there any possible physiological explanations, or could selection bias have influenced the results?*

- Response: We have comprehensively revised the chapters on “selection bias” and “associations between anisometropia and spherical ametropia”. In doing so, we have added further explanations for our results and have addressed selection bias, which could result from the structure of the study population, much more extensively than before. We have also incorporated our clinical experience into the discussion. In terms of potential physiological explanations for our results, we have added further literature and ultimately come to the conclusion that, due to the multifactorial causes of anisometropia, further research in this area on the basis of the general population is indicated in order to be able to completely rule out selection bias. However, in the discussion we also address why we nevertheless consider our results to be of high relevance.

Thank you for your time and consideration.

Sincerely,

Andreas Frings, MD

Corresponding Author

University Hospital Düsseldorf
